# Clinical benefit of high tibial osteotomy combined with the intervention of platelet-rich plasma for severe knee osteoarthritis

**DOI:** 10.1186/s13018-022-03304-0

**Published:** 2022-09-05

**Authors:** Conglei Dong, Chao Zhao, Fei Wang

**Affiliations:** Department of Orthopaedic Surgery, Third Hospital of Hebei Medical University, Ziqiang Road 139, Shijiazhuang, 050051 Hebei China

**Keywords:** High tibial osteotomy, Platelet-rich plasma, Severe knee osteoarthritis, Cartilage regeneration

## Abstract

**Purpose:**

The objective of present study was to investigate the therapeutic effects of high tibial osteotomy (HTO) combined with platelet-rich plasma (PRP) for severe knee osteoarthritis (KOA).

**Methods:**

This was a double-blinded, randomized, placebo-controlled trial. The participants were randomly divided by computerderived random charts into 3 groups: 24 participants in group A (24 knees) received a treatment option of HTO combined with PRP, 25 participants in group B (25 knees) received a treatment program of HTO combined with hyaluronic acid, and 25 participants in group C (25 knees) received a treatment method of HTO combined with normal saline (NS) (physiological control/placebo). The Western Ontario and McMaster Universities Osteoarthritis Index (WOMAC) and visual analog scale (VAS) were measured preoperatively and at the final follow-up. Status of articular cartilage was assessed according to the International Cartilage Repair Society grade and the presence of newly formed cartilaginous tissue by arthroscopy. MRI was completed of knee joint to measure the cartilaginous thickness.

**Results:**

Compared to Group B and C, the final follow-up results of knee function in Group A were significantly different (*P* < 0.001), such as the total WOMAC score 18.54 (SD 4.17), the VAS score 1.72 (SD 0.53). Cartilage regeneration of femur and tibia in Group A was observed in all patients. The cartilaginous thickness in Group A were significantly different (*P* < 0.001), such as the anterior patella femoral region 3.52 (SD 0.47), the anterior meniscal region 1.16 (SD 0.24), the posterior meniscal region 1.24 (SD 0.26) and the posterior condyle region 2.25 (SD 0.31).

**Conclusions:**

The addition of combined PRP to HTO may be a more reasonable choice to relieve knee pain and decelerate the progression of the medial KOA.

## Introduction

Osteoarthritis (OA) is the most frequent joint disorder with a worldwide increase over the past few years [1]. Knee osteoarthritis (KOA) is the most common type and is encountered in 6% of adults, with a prevalence reaching 40% in advanced age (> 70 years) [2].

KOA is a degenerative disease characterized by biochemical and its changes in articular cartilage [3]. The medial compartment is most frequently affected in knee OA due to its association with a metaphyseal varus malalignment leading to raised loading on the medial articular surface [4]. High tibial osteotomy (HTO), as a classic KOA step surgical treatment with good clinical effect, is a widely accepted procedure to treat varus alignment of the knee associated with medial compartment arthrosis/overload [5–8]. More importantly, there is increasing clinical evidence that the surface of exposed subchondral bone may be regenerated and covered with fibrous cartilage under decompression after osteotomy [9–11]. Therefore, HTO not only corrects the biomechanics of articular cartilage, but also improves the mechanical environment of cartilage regeneration, which indirectly plays a biochemical advantage to some extent.

In fact, the damaged articular cartilage has a very weak self-regeneration and repair capacity [12–15]. But the regeneration and anti-inflammatory effects of platelet-rich plasma (PRP) play an important biochemical role in cartilage repair [16–18]. At present, used in isolation or in combination intra-articular injection of PRP has been widely used in the treatment of KOA, and its clinical evaluation has been significantly improved compared with that before treatment [19–25]. Therefore, in view of the obvious biochemical advantages of PRP, this study proposed a new scheme for the clinical treatment of KOA: based on high tibial osteotomy, combined with intra-articular injection of PRP. Theoretically, PRP will play a catalytic role in the process of cartilage regeneration after HTO, and the combination of these two treatments should have an augmentation effect on the therapeutic effect of KOA. Therefore, the purpose of our study is to explore the clinical efficacy of the addition of combined PRP to HTO in the treatment of KOA.

## Materials and methods

### Patients

The study had ethical approval and all patients gave informed consent.

The inclusion criteria were: (1) medial compartment arthritis; (2) chronic joint pain > 12 months, with no significant effect of non-operative treatment; (3) X-ray evaluation of articular cartilage damage were consistent with Kllgren-Lawrence (K-L) grade 3/4; (4) arthroscopic evaluation of articular cartilage damage was consistent with International Cartilage Repair Society (ICRS) grade 3/4.

The exclusion criteria were: (1) age > 65 years old; (2) combined with severe lateral compartment arthritis or patellofemoral arthritis; (3) flexion contracture greater than 15°; (4) combined with rheumatoid arthritis, gouty arthritis, traumatic arthritis; previous history of knee surgery, physiotherapy or intra-articular injection within 6 months, anticoagulant or immunosuppressant within 3 months; accompanied by diabetes mellitus, severe cardiovascular disease, coagulation dysfunction, immune system diseases, hepatopathy, infections or tumors.

**Fig. 1:**
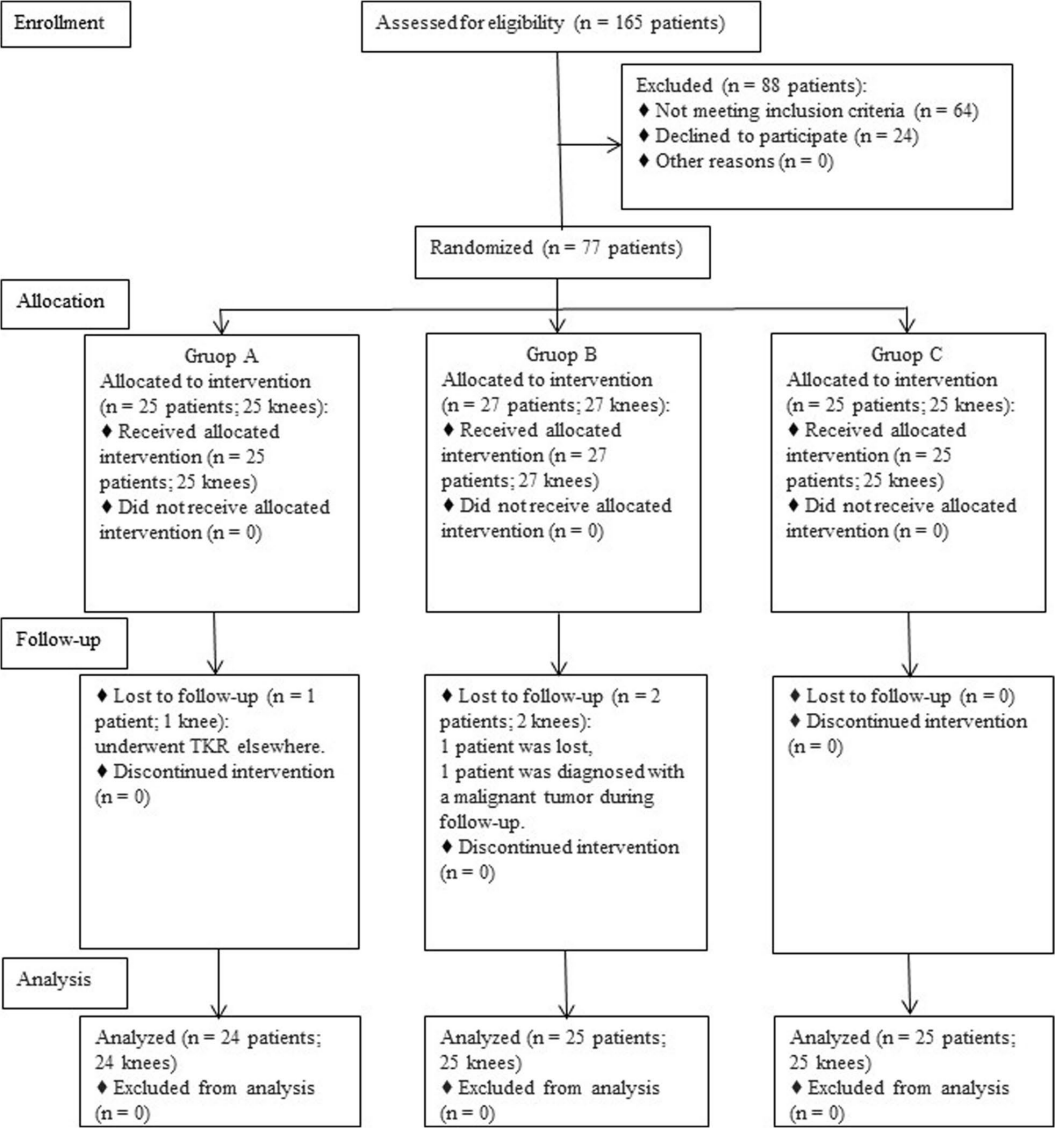
The Consolidated Standards of Reporting Trials (CONSORT) flowchart

This was a double-blinded, randomized, placebo-controlled trial with 3 groups receiving 3 different lines of treatment (1 group serving as placebo controls). The participants were randomly divided by computer derived random charts into 3 groups: 25 participants in group A (25 knees) received a treatment option of HTO combined with PRP, 27 participants in group B (27 knees) received a treatment program of HTO combined with hyaluronic acid (HA), and 25 participants in group C (25 knees) received a treatment method of HTO combined with normal saline (NS) (physiological control/placebo). Randomization ensured that the baseline characteristics of the 3 groups were comparable with age, sex, weight, height, body mass index (BMI), and K-L grade. Of the 25 patients in group A, 1 patient was excluded as he underwent total knee replacement (TKR) elsewhere; Of the 27 patients initially in group B, 1 patient was lost, and 1 patient was excluded as he was diagnosed with a malignant tumor during follow-up. The Consolidated Standards of Reporting Trials (CONSORT) flowchart showing the selection of patients is shown in Fig. 1. For clinical evaluation, the Western Ontario and McMaster Universities Osteoarthritis Index (WOMAC) score and the visual analog scale (VAS) were measured preoperatively and at the final follow-up (12 months after HTO). Initial arthroscopy was performed at the time of HTO, and a second-look arthroscopy was performed at the time of plate removal (12 months after HTO), and the status of articular cartilage was assessed according to the ICRS grade. Cartilage regeneration was also evaluated by the presence of newly formed cartilaginous tissue. In addition, MRI was completed of each joint to measure the cartilaginous thickness of the medial femoral condyle.

### Surgical procedure and postoperative management

**Fig. 2:**
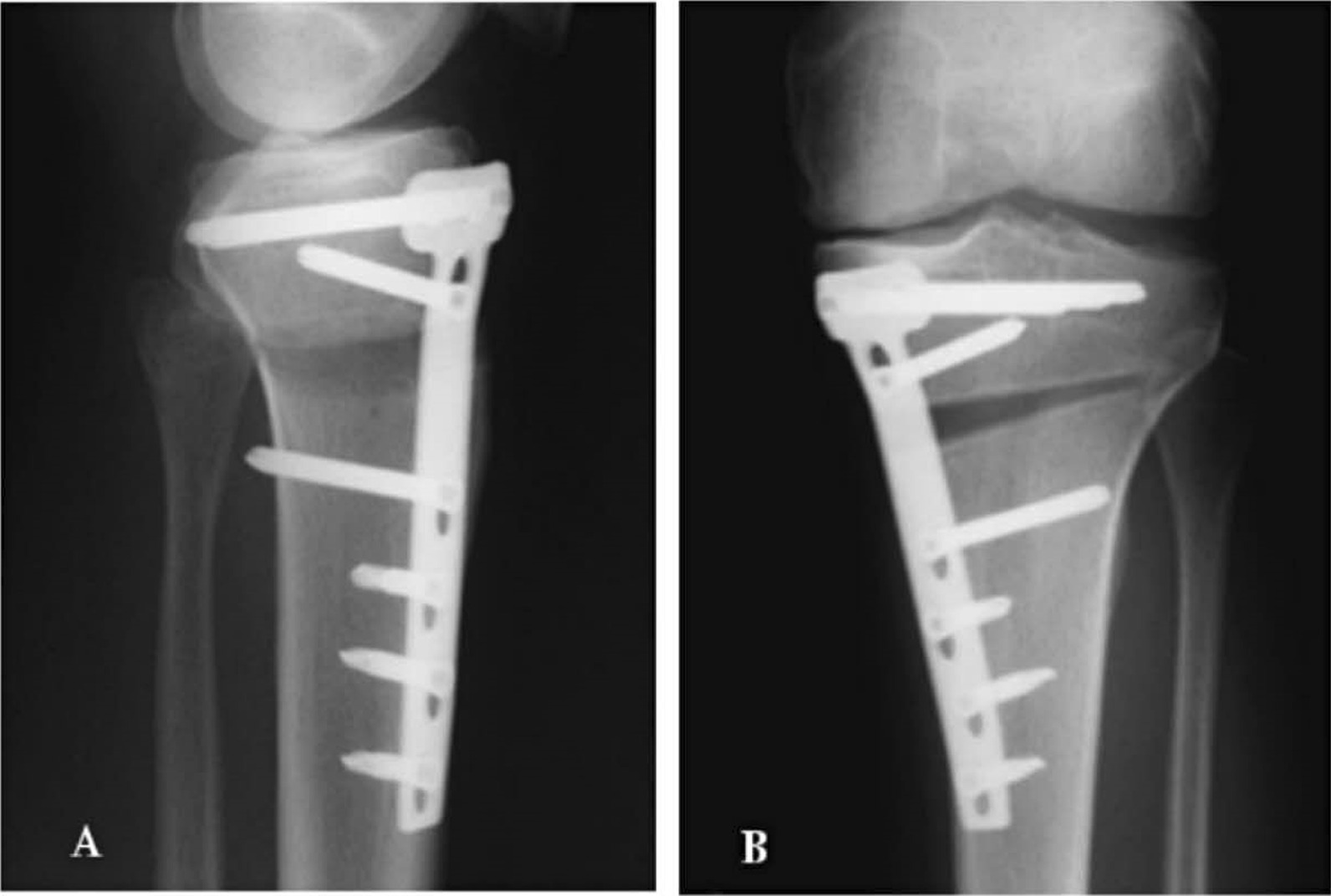
The lateral **A** and antero-posterior **B** X-ray of opening-wedge valgus HTO

An arthroscopy was routinely performed before HTO to evaluate the medial and lateral cartilage. HTO was performed using the biplanar opening-wedge technique with rigid plate fixation [4, 26] (Fig. 2). The amount of angular correction was planned preoperatively aiming to achieve 5 degrees tibiofemoral mechanical valgus in a one-leg standing radiograph postoperatively [27, 28]. All patients’ operations were performed by the same senior surgeon.

Patients started a postoperative rehabilitation program including isometric quadriceps exercise and range-of-motion exercise the day after surgery. A non-weight-bearing regimen was prescribed for 1 week, followed by partial weight-bearing exercise. The full weight-bearing exercise was permitted 4 weeks postoperatively. Casts or supportive devices were never applied.

### PRP preparation and interventional procedure

Autologous PRP was prepared according to the method of Landesberg [29]. A total of 50 mL venous blood was extracted and centrifuged 2 times to obtain about 5 mL PRP, of which 3 mL was used for intra-articular injection therapy, and the rest was used for platelet count. The mean platelet count achieved by our method was 142.14 × 10^4^/uL. The effective concentration limit of PRP in the human body is (50.30 ~ 172.90) × 10^4^/uL [30]. When the platelet concentration exceeds 180.00 × 10^4^/uL, its ability to promote tissue regeneration and repair is significantly weakened, and even shows an inhibitory effect [30, 31]. Marx et al. believe that platelet-rich plasma with a platelet concentration of about 4 ~ 5 times (120.00 ~ 150.00 × 10^4^/uL) has a positive effect on articular cartilage [32]. None of the groups knew how much blood was extracted, as they were instructed to look the other way during extraction; only 5 mL of blood was extracted in the control group and was subjected to routine testing.

The patient was placed in a supine position with the affected knee in slight flexion. Under aseptic conditions, 3 mL of autologous PRP, HA,or NS was uniformly injected into the articular cavity through the medial articular space of the patellar tendon below the patella. If there is more effusion in the articular cavity, part of the effusion can be drawn out first. After the injection, the patient was assisted to move the knee joint slowly several times and told to rest for 10 min. The treatment was first performed 1 week after HTO, which was requested again at intervals of 1 week for a total of four consecutive times. During the follow-up period, nonsteroidal anti-inflammatory drugs were not allowed, and paracetamol (dosage, 500 mg TDS) was prescribed in case of discomfort; all patients were asked to stop medications 48 h before follow-up assessment.

### Assessments

The WOMAC score is a widely used measure of a patient’s subjective assessment of pain, joint mobility and physical disability [33]. The VAS is a widely used measure of a patient’s subjective assessment of pain [24].

The grade of cartilage injury associated with degeneration was recorded pre-and post-operatively in each compartment according to the ICRS grade [34]: Grade 0, normal; Grade 1, softening of the articular cartilage and superficial lacerations and fissures; Grade 2, fragmentation and fissuring that extends < 50% of the articular cartilage thickness; Grade 3, fragmentation and fissuring that extends > 50% of the articular cartilage thickness; and Grade 4, complete loss of articular cartilage thickness. Postoperative cartilage regeneration was also evaluated using a modified classification of the macroscopic staging system described by Koshino et al. [11]: Grade 1, no regenerative change; Grade 2, white scattering with fibrocartilage; Grade 3, partial coverage with fibrocartilage; and Grade 4, even coverage with fibrocartilage. The intra and interobserver reliabilities of arthroscopic evaluation (ICRS grade and stage of articular cartilage repair) were assessed by weighted kappa coefficients.

**Fig. 3:**
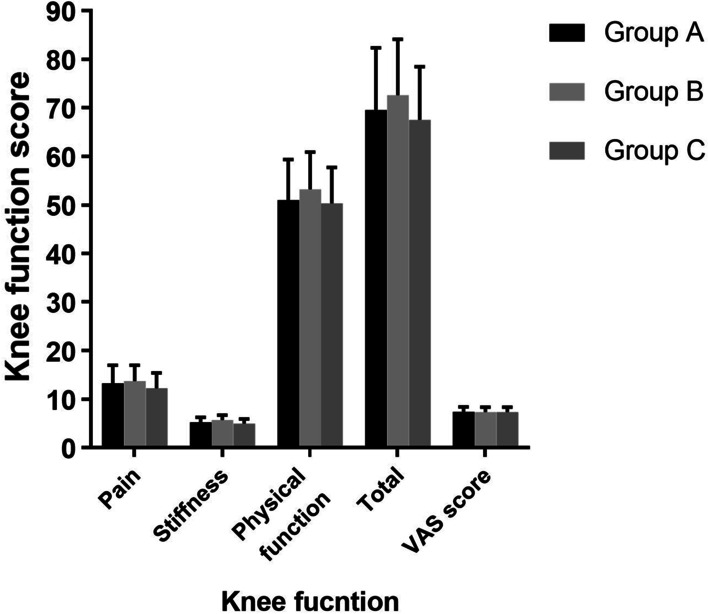
Preoperative evaluation of knee function

**Fig. 4:**
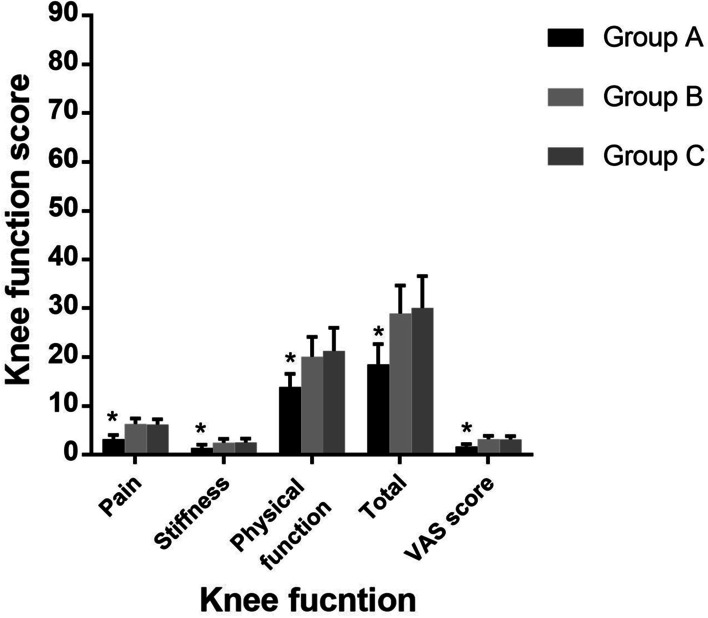
Follow-up evaluation of knee function

MRI was completed with 1.5T standard protocols of each individual joint in the coronal sagittal and transverse plane. Maximum thickness of cartilage at posterior/ meniscal and patellar level measured at mid-sagittal thru medial condyle was taken into account. The medial femoral cartilage of the affected knee was selected for measurement. Tree regions of the medial femoral condyle were identified at the anterior patella femoral, meniscal and posterior condyle levels. The point has a maximum thickness in the sagittal section passing thru the middle was identified for measurement [35]. All data were measured using Sante DICOM Viewer Free (64-bit) version 5.2 (Santesoft, Inc. Athens, Greece), which has an accuracy of 0.01° for angles and 0.01 mm for distance [36]. In order to minimize errors of measurement, all measurements were performed under the same conditions by two orthopedic surgeons. After an interval of two weeks, one measured the samples again and the intra and interobserver reliabilities were determined using intra-class correlation coefficients (ICCs) (Figs. 3 and 4).

### Statistical analysis

Our sample size was based upon an assumed study power of 80% (*β* = 0.2), a false-positive rate of 5% (*α* = 0.05), and a predicted difference of 1.5 points on our VAS (standard deviation, ± 1.5) 24. Using these parameters, and adjusting our α for multiple comparisons, we required approximately 21 patients per treatment arm.

Statistical analysis was performed using the Statistical Package for the Social Sciences (SPSS) version 16.0 (SPSS, Chicago, Illinois). The Kolmogorov–Smirnov test was used to test the normality of numerical data. Levene’s test was used to assess the homogeneity of the data. All numerical variables showed a normal distribution or equal variance. Differences for all three groups (WOMAC score, VAS score and the cartilaginous thickness of medial femoral condyle) were analyzed using the analysis of variance (ANOVA). The association of various categorical/classified data (arthroscopic evaluation, including the ICRS grade and the stage of articular cartilage repair), within the three groups was analyzed using Pearson’s chi-squared test. Numerical data are shown as mean and standard deviation, and categorical/classified data as numbers with percentages. A *p*-value of < 0.05 was considered statistically significant.

## Results

### General information and preoperative assessment of patients

**Fig. 5:**
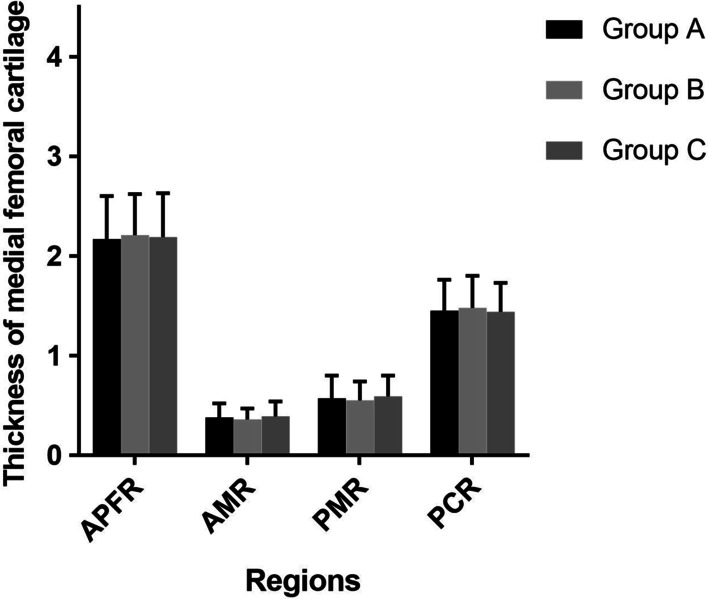
Preoperative evaluation of the thickness of medial femoral cartilage

The baseline characteristics, including age, sex, weight, height, BMI, and K-L grade, within the three groups were no statistically significant differences (*P* > 0.05, Table 1) (Tables 2, 3, 4, 5, and 6). There was no significant statistical difference in all preoperative assessments (including WOMAC score, VAS score, the cartilaginous thickness of medial femoral condyle and ICRS grade), suggesting that the data within the three groups were comparable (*P* > 0.05, Tables 2, 4, and 6 and Figs. 3 and 5). The intra and interobserver reliabilities of arthroscopic evaluation (ICRS grade and stage of articular cartilage repair) and measurement data (cartilaginous thickness of medial femoral condyle) were > 0.8.

**Table 1:** The comparison of baseline characteristics of the groups

Indexes	Group A (*n* = 24)	Group B (*n* = 25)	Group C (*n* = 25)	*p* value
Age (year)	56.64 ± 8.32	55.18 ± 7.96	56.07 ± 7.91	n.s.
Height (cm)	160.71 ± 7.39	158.93 ± 6.98	161.58 ± 8.04	n.s.
Weight (kg)	70.09 ± 8.59	68.52 ± 7.38	69.83 ± 9.45	n.s.
BMI (kg/m^2^)	27.14 ± 3.16	27.13 ± 3.04	26.75 ± 2.98	n.s.
Gender (F/M)	18/6	20/5	19/6	n.s.
KL grade, *n* (%)				n.s.
3	16 (67)	15 (60)	16 (64)	
4	8 (33)	10 (40)	9 (36)	

Age, Height, Weight, and BMI were calculated using the analysis of variance (ANOVA). The Gender and KL grade were compared by Pearson’s chi-squared test

BMI—body mass index; F—female; M—male; KL—Kllgren-Lawrence

n.s.: *p* > 0.05

**Table 2:** Preoperative evaluation of knee function

Indexes	Group A (*n* = 24)	Group B (*n* = 25)	Group C (*n* = 25)	*p* value
*WOMAC score*				
Pain	13.26 ± 3.73	13.71 ± 3.28	12.24 ± 3.17	n.s.
Stiffness	5.26 ± 1.02	5.70 ± 1.04	5.53 ± 0.96	n.s.
Physical function	50.98 ± 8.35	53.22 ± 7.68	50.33 ± 7.37	n.s.
Total	69.54 ± 12.83	72.63 ± 11.49	67.55 ± 10.94	n.s.
VAS score	7.43 ± 0.98	7.35 ± 1.03	7.36 ± 1.01	n.s.

WOMAC score and VAS score were calculated using the analysis of variance (ANOVA)

*WOMAC*—Western Ontario and McMaster Universities Arthritis Index; *VAS*—visual analog scale

n.s.: *p* > 0.05

**Table 3:** Follow-up evaluation of knee function

Indexes	Group A (*n* = 24)	Group B (*n* = 25)	Group C (*n* = 25)
*WOMAC score*			
Pain	3.22 ± 0.85*	6.35 ± 1.14	6.22 ± 1.09
Stiffness	1.43 ± 0.69*	2.53 ± 0.75	2.58 ± 0.77
Physical function	13.89 ± 2.70*	20.08 ± 4.11	21.30 ± 4.74
Total	18.54 ± 4.17*	28.96 ± 5.75	30.10 ± 6.52
VAS score	1.72 ± 0.53*	3.24 ± 0.67	3.21 ± 0.64

WOMAC score and VAS score were calculated using the analysis of variance (ANOVA)

*WOMAC*—Western Ontario and McMaster Universities Arthritis Index; *VAS*—visual analog scale

*Significant difference compared with the placebo controls (Group C)

**Table 4:** Preoperative evaluation of the thickness of medial femoral cartilage

Regions	Group A (*n* = 24)	Group B (*n* = 25)	Group C (*n* = 25)	*p* value
APFR	2.17 ± 0.43	2.21 ± 0.41	2.19 ± 0.44	n.s.
AMR	0.38 ± 0.14	0.36 ± 0.11	0.39 ± 0.15	n.s.
PMR	0.57 ± 0.23	0.55 ± 0.19	0.59 ± 0.21	n.s.
PCR	1.45 ± 0.31	1.48 ± 0.32	1.44 ± 0.29	n.s.

APFR, AMR, PMR, and PCR were calculated using the analysis of variance (ANOVA)

*APFR*—anterior patella femoral region; *AMR*—anterior meniscal region; *PMR*—posterior meniscal region; *PCR*—posterior condyle region

n.s.: *p* > 0.05

**Table 5:** Follow-up evaluation of the thickness of medial femoral cartilage

Regions	Group A (*n* = 24)	Group B (*n* = 25)	Group C (*n* = 25)
APFR	3.52 ± 0.47*	2.78 ± 0.42	2.81 ± 0.45
AMR	1.16 ± 0.24*	0.76 ± 0.11	0.81 ± 0.13
PMR	1.24 ± 0.26*	0.82 ± 0.12	0.85 ± 0.14
PCR	2.25 ± 0.31*	1.89 ± 0.28	1.91 ± 0.27

APFR, AMR, PMR, and PCR were calculated using the analysis of variance (ANOVA)

*APFR*—anterior patella femoral region; *AMR*—anterior meniscal region; *PMR*—posterior meniscal region; *PCR*—posterior condyle region

*Significant difference compared with the placebo controls (Group C)

**Table 6:** Preoperative evaluation of ICRS grade in articular cartilage

ICRS grade, *n* (%)	Group A (*n* = 24)	Group B (*n* = 25)	Group C (*n* = 25)	*p* value
MFC				n.s.
3	15 (63)	16 (64)	17 (68)	
4	9 (37)	9 (36)	8 (32)	
MTC				n.s.
3	16 (67)	15 (60)	16 (64)	
4	8 (33)	10 (40)	9 (36)	

The ICRS grade was compared by Pearson’s chi-squared test

*ICRS*—International Cartilage Repair Society grade; *MFC*—medial femoral condyle; *MTC*—medial tibial condyle

n.s.: *p* > 0.05

### Follow-up assessment of clinical outcomes

Compared with preoperative evaluation, the final follow-up results of WOMAC score and VAS score within the three groups were significantly improved (*P* < 0.001). At the final follow-up, these parameters were not significantly different between Group B and C, such as the Pain (Group B, 6.35 (SD 1.14); Group C, 6.22 (SD 1.09); *p* > 0.05), the Stiffness (Group B, 2.53 (SD 0.75); Group C, 2.58 (SD 0.77); *p* > 0.05), the Physical function (Group B, 20.08 (SD 4.11); Group C, 21.30 (SD 4.74); *p* > 0.05), the Total score (Group B, 28.96 (SD 5.75); Group C, 30.10 (SD 6.52); *p* > 0.05) and the VAS score (Group B, 3.24 (SD 0.67); Group C, 3.21 (SD 0.64); *p* > 0.05) (Table 3 and Fig. 4). But compared to Group B and C, the final follow-up results of Group A were significantly different (*P* < 0.001), such as the Pain 3.22 (SD 0.85), Stiffness 1.43 (SD 0.69), the Physical function 13.89 (SD 2.70), the Total score 18.54 (SD 4.17) and the VAS score 1.72 (SD 0.53) (Table 3 and Fig. 4).

### Follow-up assessment of articular cartilage

**Fig. 6:**
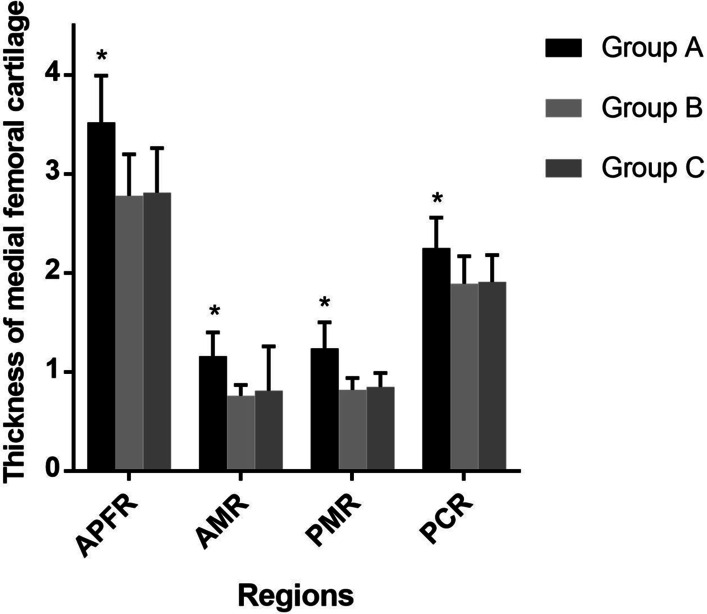
Follow-up evaluation of the thickness of medial femoral cartilage

Compared with preoperative evaluation, the final follow-up results of cartilaginous thickness of medial femoral condyle within the three groups were significantly improved (*P* < 0.001). At the final follow-up, these parameters were not significantly different between Group B and C, such as the APFR (Group B, 2.78 (SD 0.42); Group C, 2.81 (SD 0.45); *p* > 0.05), the AMR (Group B, 0.76 (SD 0.11); Group C, 0.81 (SD 0.13); *p* > 0.05), the PMR (Group B, 0.82 (SD 0.12); Group C, 0.85 (SD 0.14); *p* > 0.05) and the PCR (Group B, 1.89 (SD 0.28); Group C, 1.91 (SD 0.27); *p* > 0.05) (Table 5 and Fig. 6). But compared to Group B and C, the final follow-up results of Group A were significantly different (*P* < 0.001), such as the APFR 3.52 (SD 0.47), the AMR 1.16 (SD 0.24), the PMR 1.24 (SD 0.26) and the PCR 2.25 (SD 0.31) (Table 5 and Fig. 6).

Preoperative and postoperative assessments of cartilage damage using the ICRS grade are summarized in Tables 6 and 7. Cartilage status was significantly improved in both the MFC and the MTC after HTO (*P* < 0.001). At the final follow-up, the status of cartilage damage was not significantly different between Group B and C (*P* > 0.05); But compared to Group B and C, the cartilage status of Group A was significantly different (*P* < 0.001), whose cartilage damage was significantly reduced. Evaluation of cartilage regeneration by the presence of newly formed cartilaginous tissue at second-look arthroscopy is summarized in Table 8. At the final follow-up, cartilage regeneration was more obvious in Group A compared with Group B and C (*P* < 0.001). Articular cartilage repair of the femur in Group A was observed in all patients (100%), where 6 knees (25%) showed Grade 2 regeneration, 10 knees (42%) showed Grade 3, and 8 knees (33%) showed Grade 4 regeneration. Articular cartilage repair of the tibia in Group A was also observed in all patients (100%), where 8 knees (33%) showed Grade 2 regeneration, 11 knees (46%) showed Grade 3, and 5 knees (21%) showed Grade 4 regeneration.

**Table 7:** Follow-up evaluation of ICRS grade in articular cartilage

ICRS grade, *n* (%)	Group A (*n* = 24)	Group B (*n* = 25)	Group C (*n* = 25)
*MFC*			
1	4 (17)*	0 (0)	0 (0)
2	13 (54)*	10 (40)	9 (36)
3	7 (29)*	12 (48)	14 (56)
4	0 (0)*	3 (12)	2 (8)
*MTC*			
1	4 (17)*	0 (0)	0 (0)
2	11 (46)*	8 (32)	7 (28)
3	9 (37)*	13 (52)	15 (60)
4	0 (0)*	4 (16)	3 (12)

The ICRS grade was compared by Pearson’s chi-squared test

*ICRS*—International Cartilage Repair Society grade; *MFC*—medial femoral condyle; *MTC*—medial tibial condyle

*Significant difference compared with the placebo controls (Group C)

**Table 8:** Follow-up evaluation of articular cartilage regeneration

Presence of newly formed cartilagenous tissue, *n* (%)	Group A (*n* = 24)	Group B (*n* = 25)	Group C (*n* = 25)
*MFC*			
1	0 (0)*	3 (12)	2 (8)
2	6 (25)*	11 (44)	12 (48)
3	10 (42)*	9 (36)	10 (40)
4	8 (33)*	2 (8)	1 (4)
*MTC*			
1	0 (0)*	4 (16)	3 (12)
2	8 (33)*	11 (44)	13 (52)
3	11 (46)*	9 (36)	8 (32)
4	5 (21)*	1 (4)	1 (4)

The grade of articular cartilage regeneration was compared by Pearson’s chi-squared test

*MFC*—medial femoral condyle; *MTC*—medial tibial condyle

*Significant difference compared with the placebo controls (Group C)

## Discussion

The main finding of this study was that the addition of combined PRP to HTO may be a more reasonable choice to relieve knee pain and decelerate the progression of the medial KOA.

Compared with pre-treatment, the knee function scores (WOMAC and VAS score) of all patients within the 3 groups were significantly improved, which is closely related to the obvious biomechanical advantages of HTO. This is achieved by shifting the mechanical axis towards the lateral compartment with a slight over-correction to off-load the medial compartment, resulting in relieving knee pain and decelerating the progression of the medial OA [37–39]. The multicentric follow-up results of Floerkemeier [4] revealed the good-to-excellent patient-reported functional outcome after valgus HTO in varus osteoarthritis with a mean OKS of 43, even in older patients with a higher degree of medial cartilage damage. For the severe medial OA of K-L grade 3/4, Schuster P [40] noted that the knee function score was high at a 10-year follow-up after HTO, and the 10-year survival rate could reach 81.7%. In our study, both X-ray and arthroscopic evaluations of articular cartilage damage were serious preoperatively, but the knee function scores were significantly improved after HTO, which was consistent with the above conclusions. Overall, the mid- and long-term results of HTO are cited as good, with reduction in pain and facilitation of increased activity, particularly with the development of specific fixation devices and osteotomy techniques [38, 41]. A meta-analysis [42] indicated that the 10-year survival rate of more than 600 cases after HTO could reach 91.5% and the lifelong revision rate of osteotomy was about 35%, so most patients did not even need joint replacement.

**Fig. 7:**
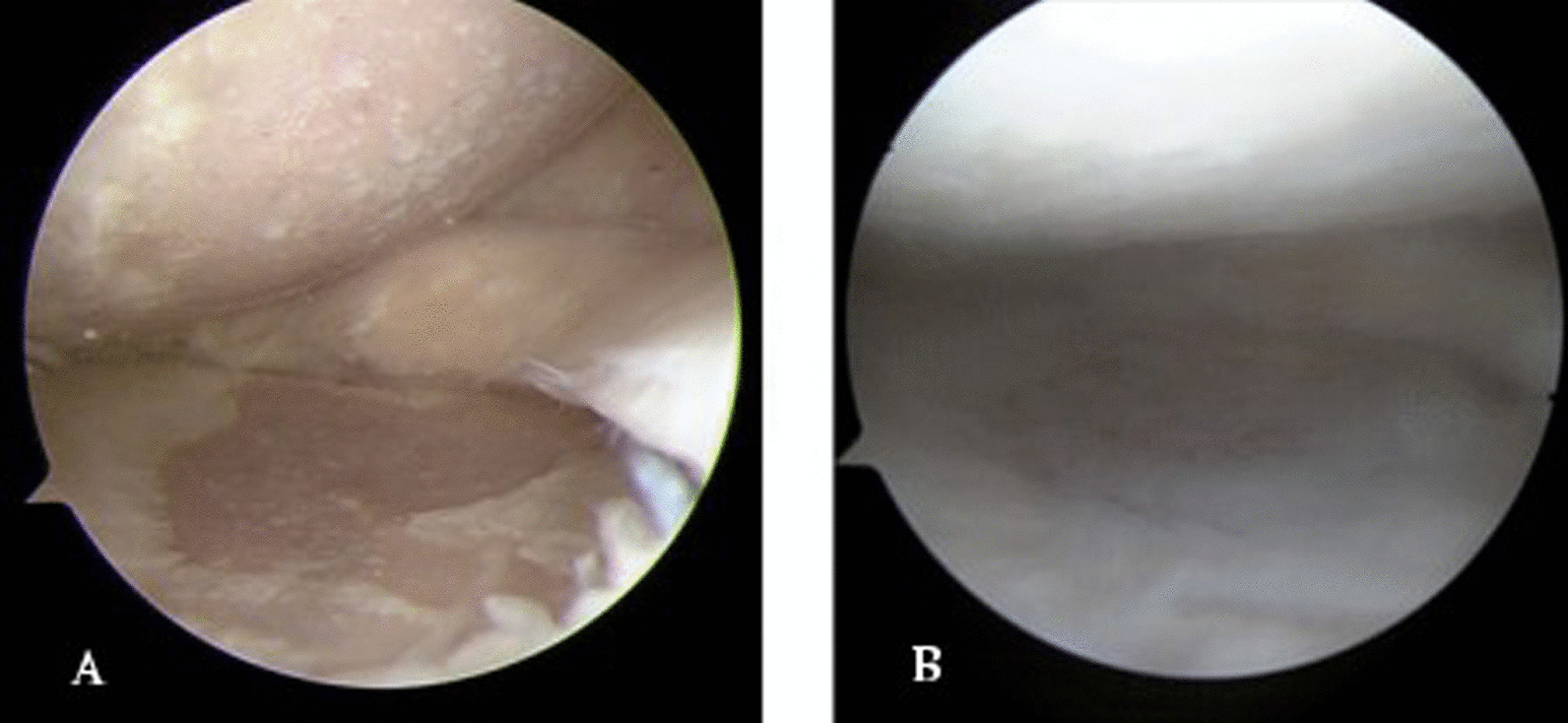
**A** arthroscopic view at initial surgery and **B** second-look arthroscopy in Group A

**Fig. 8:**
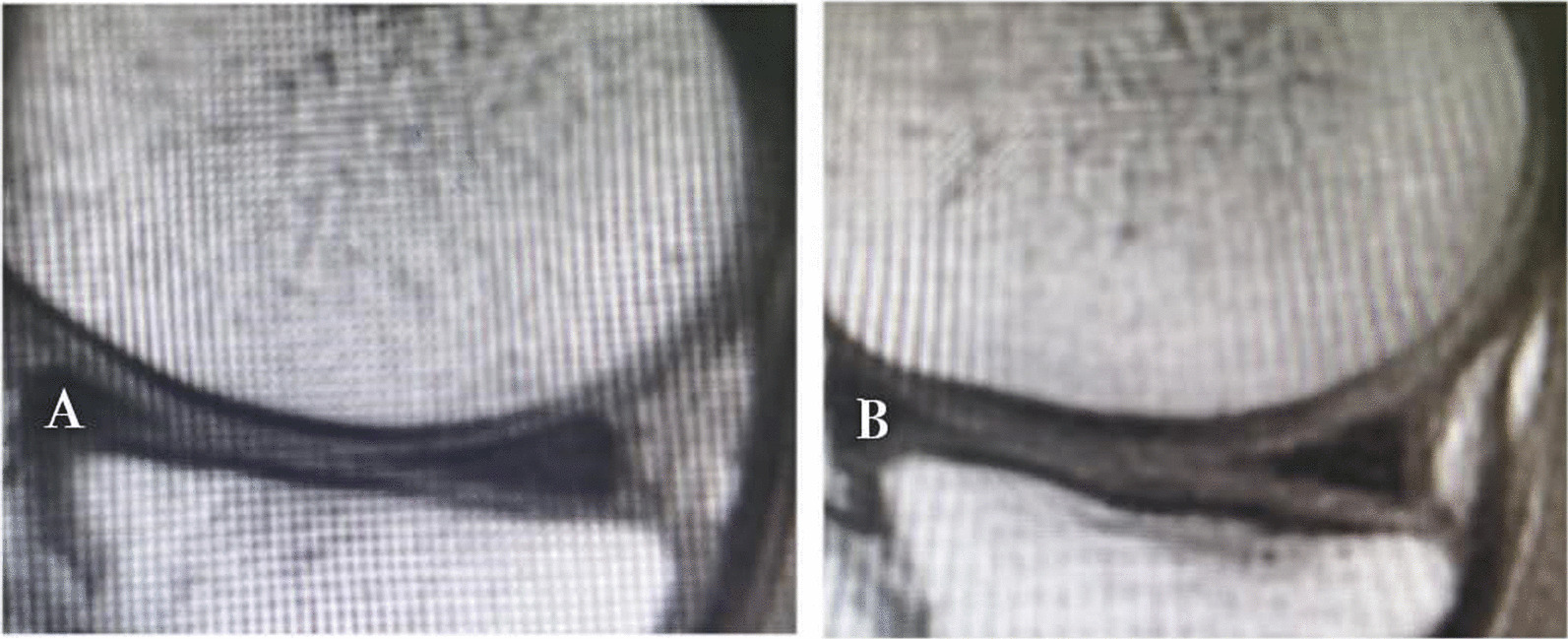
**A** MRI baseline and **B** 12 months follow-up. Improvement at all levels in Group A

The decompression of the medial compartment after HTO could provide a good mechanical environment for cartilage regeneration [10]. It has been reported in the relevant literature that even in the cases of full-thickness cartilage damage, up to 78% of the patients will show the phenomenon of cartilage regeneration after osteotomy [9–11, 40]. But the ability of articular cartilage to repair itself is very limited. When ideal correction was obtained, the ulcerated lesion was thoroughly covered with fibrous and membranous tissue at least 1.5–2 years after osteotomy [43–46]. Accordingly, in order to enhance and accelerate the process of cartilage regeneration after osteotomy [47], PRP was combined in this study to play a catalytic role similar to that of an enzyme. A high concentration of platelet-derived growth factors in PRP can not only stimulate chondrocyte proliferation and matrix secretion,but also reduce the expression of inflammatory factors and apoptosis of chondrocytes [16–18]. Furthermore, relevant research has shown that the therapeutic effect of PRP can be maintained for at least 12 months [19, 20, 44]. However, HA was only aimed at the relief of pain and inflammation, which could not reduce the degeneration and destruction of articular cartilage [35], and its curative effect gradually deteriorated within 1–6 months after treatment and tended to the pre-treatment state [23]. In our study, cartilage regeneration in Group A was the most evident both by MRI measurements and arthroscopic evaluation (Figs. 7 and 8), and the phenomenon in Group C was completely due to the decompression of the medial compartment after osteotomy, with no significant statistical difference compared to Group B, which was reasonable according to the above conclusions.

However, according to these studies which examined PRP effectiveness on the knee joint, better results were achieved in patients with a low degree of cartilage degeneration, and the effect decreased when degeneration was worse [45–48]. As joint degeneration increases, factors such as a decrease in viable cells, muscle function deficiency, joint instability due to increased ligament laxity, decrease in anabolic response to growth factors, loss of chondrocyte and thinning of cartilage plate may diminish the effectiveness of PRP [49]. Despite poorer results, patients with advanced OA still benefit from PRP. In a comparative study of PRP and HA in grade 3 knee OA, the PRP group showed significantly better results after 6 months and the worse results were observed in HA-treated subjects [23]. And Calis et al. showed that PRP administered three times at weekly intervals to patients with grade 3 and 4 knees OA reported improvements in their quality of life, and reduced levels of pain, and had increased cartilage thickness as measured by ultrasonography at the 6-month follow-up [50]. Therefore, the knee function scores and cartilage regeneration in Group A were better compared with Group B and C in our study, attributing to the combined effects of HTO biomechanical advantages and PRP biochemical advantages.

There are some limitations of the present study. First, the number of cases included in this study was small. Second, the follow-up period was short. Third, the present study did not explore the correlation between clinical outcomes and the extent of cartilage regeneration. Fourth, control for confounding factors in study and control patients remained inadequate. Some studies [51–55] have suggested, cartilage regeneration does not always relate to clinical outcomes. However, it is believed that long-term follow-up care in a larger number of cases is necessary to accurately evaluate the clinical significance of the articular cartilage repair after treatment.

Despite the limitations mentioned above, it is believed that this study differs from other studies because it proposes a new scheme for the clinical treatment of KOA: high tibial osteotomy combined with PRP intervention, which owns the combined effects of HTO biomechanical advantages and PRP biochemical advantages. Furthermore, in the present study of medial KOA with a higher degree of cartilage damage, the Group A of HTO combined PRP shows significantly better results after 12 months compared to the treatment of HTO combined HA or NS.

In conclusion, good clinical outcomes and cartilage regeneration with advanced OA are induced after opening-wedge valgus HTO, which is affected by postoperative limb alignment. Furthermore, better follow-up results are obtained for HTO with PRP intervention than for HTO alone, which is affected by the obviously biochemical advantages of PRP. Therefore, the combination therapy of HTO with PRP may be a more reasonable choice to relieve knee pain and decelerate the progression of the medial KOA.

## Data Availability

All of the data and materials are available.

## Abbreviations

HTO: High tibial osteotomy
PRP: Platelet-rich plasma
KOA: Knee osteoarthritis
HA: Hyaluronic acid
NS: Normal saline
OA: Osteoarthritis
K-L: Kllgren-Lawrence
ICRS: International Cartilage Repair Society
BMI: Body mass index
TKR: Total knee replacement
CONSORT: Consolidated Standards of Reporting Trials
WOMAC: Western Ontario and McMaster Universities Osteoarthritis Index
VAS: Visual analog scale
ICCs: Intra-class correlation coefficients
SPSS: Statistical Package for the Social Sciences
ANOVA: Analysis of variance
APFR: Anterior patella femoral region
AMR: Anterior meniscal region
PMR: Posterior meniscal region
PCR: Posterior condyle region
MFC: Medial femoral condyle
MTC: Medial tibial condyle
